# Overoxidation of Intrinsically Conducting Polymers †

**DOI:** 10.3390/polym14081584

**Published:** 2022-04-13

**Authors:** Rudolf Holze

**Affiliations:** 1 Institut für Chemie, Chemnitz University of Technology, D-09107 Chemnitz, Germany; rudolf.holze@chemie.tu-chemnitz.de; 2 Institute of Chemistry, Saint Petersburg State University, 199034 St. Petersburg, Russia; 3 State Key Laboratory of Materials-Oriented Chemical Engineering, School of Energy Science and Engineering, Nanjing Tech University, Nanjing 211816, China

**Keywords:** intrinsically conducting polymer, ICP, radicals, polyaniline, polypyrrole, polythiophene, oxidation

## Abstract

Intrinsically conducting polymers may undergo significant changes of molecular structure and material properties when exposed to highly oxidizing conditions or very positive electrode potentials, commonly called overoxidation. The type and extent of the changes depend on the experimental conditions and chemical environment. They may proceed already at much lower rates at lower electrode potentials because some of the processes associated with overoxidation are closely related to more or less reversible redox processes employed in electrochemical energy conversion and electrochromism. These changes may be welcome for some applications of these polymers in sensors, extraction, and surface functionalization, but in many cases, the change of properties affects the performance of the material negatively, contributing to material and device degradation. This report presents published examples, experimental observations, and their interpretations in terms of both structural and of material property changes. Options to limit and suppress overoxidation are presented, and useful applications are described extensively.

## 1. Introduction

The term “oxidation” is firmly established in chemistry with different meanings in various fields [1,2]. In many fields, oxidation means a reaction with oxygen resulting in the formation of compounds such as metal oxides or carbon dioxide. Overoxidation in a general sense means the oxidation of a substrate (ion, molecule, polymer, etc.) to a higher state of oxidation than intended or desired; for typical examples, see [3,4]. In a more basic approach, it means the removal of electrons from a substrate associated with their transfer to an oxidizing agent (oxidant), e.g., dioxygen. In electrochemistry, it is always the removal of an electron (or several) and its transfer either to an electrode (an electron-conducting material called an anode) or to a mediator, which transfers this electron in turn to an electrode. Intrinsically conducting polymers (ICPs) are formed in most reported procedures by the oxidation of a monomer yielding a reactive intermediate (e.g., a radical cation), which subsequently undergoes further reactions, such as dimerization, etc. This applies both to chemical and electrochemical polymerization. There are numerous reports on the mechanisms, intermediates, and further details of the associated processes [5,6,7,8,9].

The term “intrinsically conducting polymer” names a class of mostly organic oligomeric or polymeric materials that show electronic conductivity because of mobile charge carriers capable of moving along conjugated segments and hopping between such segments of the polymer. These charge carriers are created by oxidation, i.e., the formation of a radical cation by the removal of an electron. This cation is frequently called a polaron. For charge compensation, anions from the electrolyte solution move into the ICP; in the simplest case, during oxidation with iodine, the formed iodide stays in the ICP. As an alternative, cations (e.g., protons) can move out. The ingress of anions is frequently called “doping” in a slightly stretched interpretation of the meaning of this term in solid state and semiconductor physics, where doping means the replacement of one kind of atom by atoms of another kind with a different number of valence electrons at an extremely low concentration. Consequently, the term is not used in this communication. Unfortunately and somewhat misleadingly, materials afforded also with electronic conductivity by the addition of an electronically conducting material, such as metal fibers or carbon powder, to an insulating polymer are called simply conducting polymers.

Because of this amazing merge of the typical properties of an organic material with those of a metal, i.e., a very inorganic material, ICPs have sometimes been called synthetic metals. Unfortunately, the latter term has also been applied to a class of crystalline materials, charge transfer complexes (e.g., *N*,*N*′-dicyanonaphthaquinonediimine (DCNNI) and tetrathiafulvalene (TTF)), resulting in some confusion [10,11].

Overoxidation (infrequently and for unknown reasons, the spelling over-oxidation can be found also) of an ICP may happen already during preparation by both chemical and electrochemical oxidation, as well as during the rarely applied enzymatic polymerization [12]. Used chemical oxidants, as well as the electrode potential needed to form reactive intermediates by oxidation of the respective monomer, are generally strong or high enough to oxidize and sometimes even overoxidize the already formed oligo- and polymer products. This was convincingly demonstrated using a radiotracer method for polyaniline (PANI) [13]. Highly oxidized oligo- and, in particular, polymer products (e.g., the pernigraniline form of PANI) are susceptible to chemical reactions with constituents of the environment. In the case of an ICP exposed to an electrolyte solution or a polymerization procedure using a solution containing a solvent, chemical oxidant, or dissolved supporting electrolyte, there are plenty of reactants capable of starting, e.g., bond splitting or a nucleophilic attack at the cationic sites of the oligo- or polymer-yielding substitution products. ICPs exposed to air or other gaseous environments may also undergo overoxidation in cases when suitable oxidants are available. This resulted in the terminology “chemically oxidative degradation” in an early study of the decrease in electric conductance of polypyrrole (PPy) and some composites containing it upon exposure to air, in particular, at elevated temperatures [14]. Because frequently negative effects are associated with overoxidation (in particular the undesired ones), concerns regarding its implications for practical uses of ICPs have been stated [15]. Taking the term “corrosion” in its general meaning beyond the corrosion of metals, the degradation of ICPs frequently associated with overoxidation has also been called corrosion [16,17].

Organizing the reported results and conclusions related to negative effects, i.e., degradation or decreasing performance capabilities, found across a very wide-reaching field is attempted by presenting first experimental observations and their interpretations grouped according to the well-known parent polymers PANI, polypyrrole (PPy), and polythiophene (PTh). Beyond overoxidation, there are numerous other processes also causing material, function, and device degradation; the interested reader might wish to examine the published literature for reports on the specific type of degradation of interest. The present report focuses on overoxidation only. In a following chapter, applications of overoxidation in the pursuit of materials, e.g., sensors or other functional materials, are discussed.

## 2. Experimental Observations

In electrochemical studies and applications of ICPs, overoxidation may occur when the electrode potential is moved to be more positive than a specific value. Depending on the type of ICP and the electrolyte solution, further experimental details may be important, too (for examples, see [18]). Overoxidation may happen already during the electrochemical formation of an ICP; again, the consideration of said experimental conditions applies. Details of the electrolyte solution, particularly the type of solvent, the acidity, the concentration of the electrolyte, and the type of anion, are important. The anion of the electrolyte has attracted attention at various stages of polymer formation and behavior [19]: there are reports for various ICPs on anion-specific effects during formation [20,21], during redox processes, and during overoxidation [19]. These observations were extended to anion-specific effects relevant for specific applications, as in battery electrode materials [22]. Overoxidation may also be caused by chemical treatment with, for example, hypochlorite solutions, causing tremendous decreases in the electronic conductivity of PEDOT:PSS by ten orders of magnitude, which was attributed to the chemically induced interruption of percolation pathways for electronic conduction [23]. Further chemical procedures are reported below.

Because in many cases the effects of overoxidation are detrimental (e.g., causing lower electronic conductivity [24] or lower charge storage capability), researchers have looked into possibilities for inhibiting overoxidation or for at least ameliorating its effects. These reports are collected in a section following the ICP-specific overview below. Finally, in a later chapter, the benefits of overoxidation, i.e., the practical exploitation, are presented and discussed.

### 2.1. PANI

**Figure 1:**
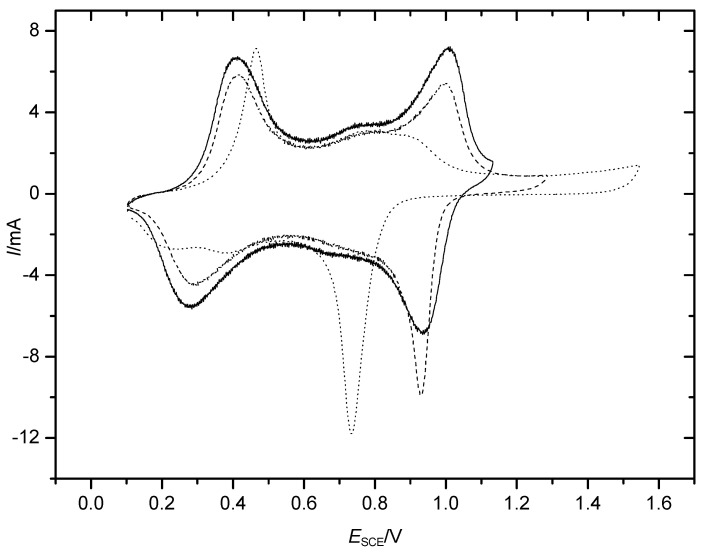
CV of a polyaniline film on a platinum electrode in an aqueous solution of 1 **M** HlO_4_ during successive overoxidation (for details, see also [26]).

A typical CV recorded with a film of PANI in an aqueous solution of 1 **M** HClO_4_ with various increasing upper electrode potential limits is shown in Figure 1. After an excursion to *E*_RHE_ = 1.5 V, the second oxidation peak associated with the transition from the emeraldine to the pernigraniline form (for details, see [25]) disappeared, and a reduction peak around *E*_RHE_ = 0.74 V appeared. It did not have an oxidation counterpart.

**Figure 2:**
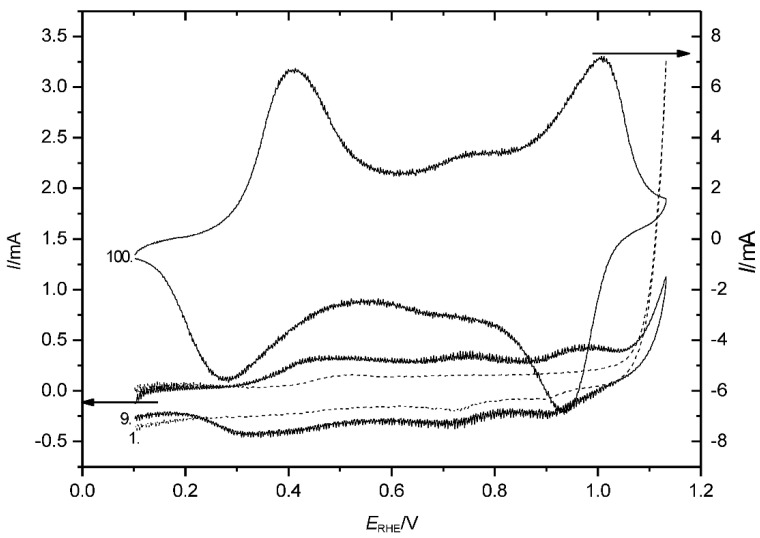
CVs of a platinum electrode in an aqueous solution of 0.1 **M** aniline in 1 **M** HClO_4_, nitrogen purged, d*E*/d*t* = 0.1 V·s^−1^; cycle numbers indicated (for details see also [26]).

At this electrode potential, a very weak current wave (Figure 2) can be observed already during polymerization, even without excursions of the positive potential limit into ranges wherein overoxidation may be expected. With radiotracer studies, this assumption of an additional electrochemical process was supported [13].

This peak pair is frequently named the “middle-potential peaks” and has been attributed to a soluble molecular quinodiimine or quinone redox system [19,27,28,29,30]. The reacting species are formed by the degradation of PANI by, for example, overoxidation and hydrolysis (see also reports on electrochemical oxidative PANI degradation [31,32,33,34,35]). During polymerization, the redox processes of dimeric species formed in the first radical reaction were suggested as a further explanation of this redox peak pair [36].

**Figure 3:**
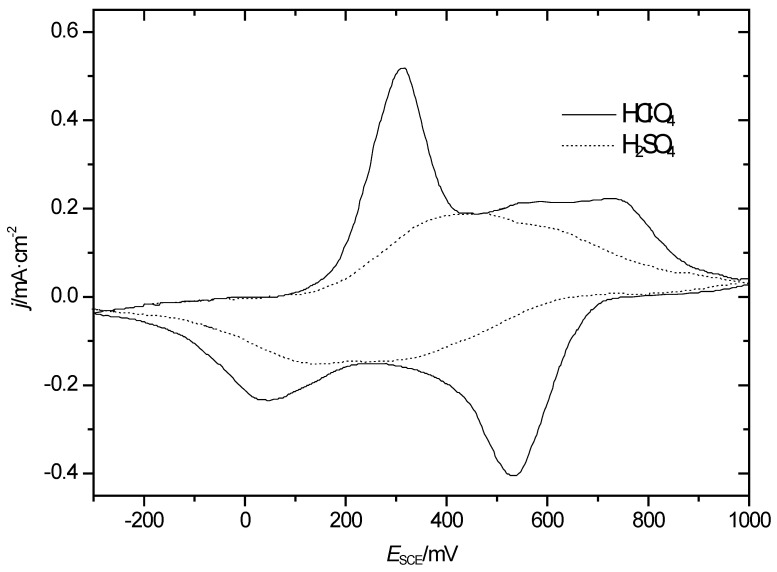
Cyclic voltammograms (CVs) of a polyaniline film on a gold electrode after a measurement series with an upper limit of *E*_SCE_ = 1000 mV in 1 **M** HClO_4_ and 0.5 **M** H_2_SO_4_ recorded at d*E*/d*t* = 0.1 V·s^−1^ (for details, see [19]).

The influences of the chemical identity of the anion and its concentration on the properties and behavior of an ICP, including overoxidation, have been addressed before. There are actually several anion-specific effects: the influence during formation (resulting in smooth or highly porous morphologies [29,30]) on the rate of formation [37]; the influence during redox processes (frequently called doping) [38,39]; and, finally, the influence during overoxidation [19,37]. As a typical example of the latter effect relevant in the present context, the CVs of PANI in two different electrolyte solutions are shown in Figure 3 after the polymer was exposed in previous CVs to electrode potentials as high as *E*_SCE_ = 1000 mV (see also Figure 1 above). In the presence of HClO_4_, changes of the first redox peak pair were small, and the second oxidation peak was less pronounced, but still the associated redox process continued. A quite different picture emerged in the presence of H_2_SO_4_.

The CV was substantially different, and a two-step process was less pronounced.

**Figure 4:**
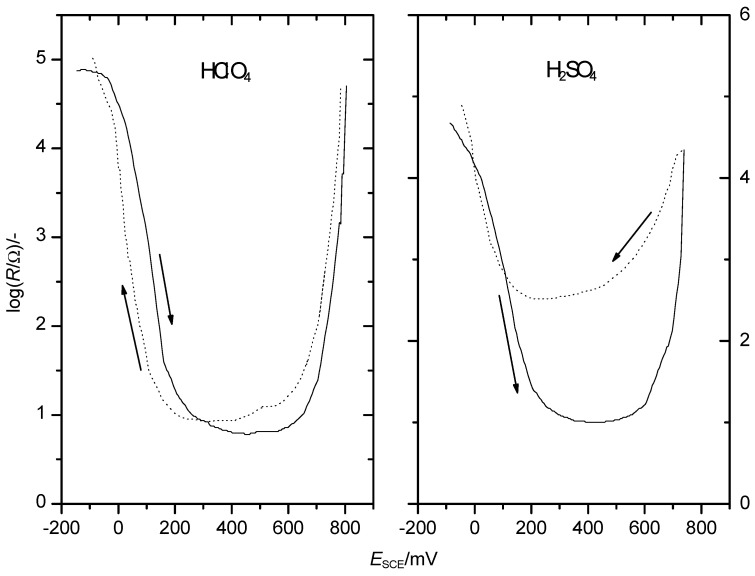
Resistance vs. electrode potential displays for a PANI film on a gold double-band electrode after a measurement series with an upper limit of *E*_SCE_ = 1000 mV in 1 **M** HClO_4_ and 0.5 **M** H_2_SO_4_ rerecorded at d*E*/d*t* = 0.1 V·s^−1^ (for details, see [19]).

The difference visible already in the CVs is also reflected in the diagrams of film resistance as a function of electrode potential shown in Figure 4.

In the presence of sulphuric acid, the resistance minimum was higher by almost two orders of magnitude. This suggests significant changes of the polymer affecting electronic conduction.

**Figure 5:**
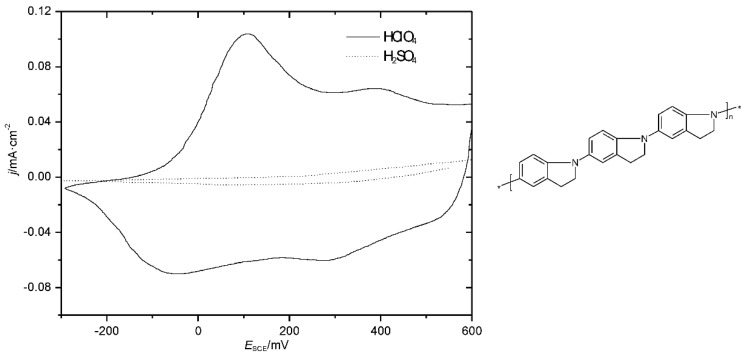
Cyclic voltammograms of a polyindoline film on a gold electrode after a measurement series with an upper limit of *E*_SCE_ = 1000 mV in 1 **M** HClO_4_ and 0.5 **M** H_2_SO_4_ recorded at d*E*/d*t* = 0.1 V·s^−1^ (for details, see [19]).

In a related study of polyindolines, CVs as displayed in Figure 5 were obtained.

**Figure 6:**
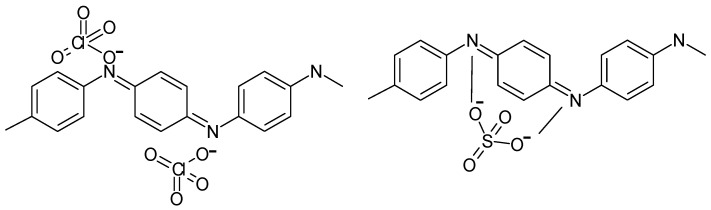
Schematic illustration of the different shielding capabilities of perchloric and sulphuric acid anions with PANI as an example.

Whereas with perchloric acid, two redox processes can still be discerned, with sulphuric acid, the redox activity of the polymer film was almost completely absent. The obviously better shielding capability of the perchlorate anions against the nucleophilic attacks of hydroxyl ions at the bipolaronic nitrogen atom sites was illustrated by the authors, as shown in Figure 6. Possibly, authors elsewhere suggesting the existence of complexes between anions and the protonated polymer chain had similar interactions in mind [37].

In a study of the stability and degradation of PANI coatings used as corrosion protection in aqueous acidic environments, dissolved benzoquinone was identified as a hydrolysis product (as already mentioned above concerning the cause of the middle-potential peak), whereas the remaining film showed properties distinctly different from those of the pristine film [40].

PANI electrodeposited potentiostatically at *E*_Ag/AgCl_ = 1 V and potentiodynamically with an upper potential limit of *E*_Ag/AgCl_ = 1.1 V on conductive textiles first coated with PPy showed large charge storage capability but, also, overoxidation with material prepared with the dynamic approach [41]. Given the sensitivity of PANI in said potential region, the findings do not come as a surprise [42]. In a study of PANI electropolymerized on the aluminum alloy 6061-T6 as an interlayer by the procedures of the previous example, the balance between the upper potential limit and deposition potential, rate of formation, and noticed overoxidation was examined [43].

The electrodeposition of PANI by a potentiostatic pulse procedure has been proposed with claims regarding fast formation and advantageous properties of the obtained ICP [42,44,45]. When the upper potential limit was moved into a region wherein presumably overoxidation could occur, the rate of PANI formation dropped significantly because the activating effect of the pulse program on PANI formation vanished [45]. Possibly, this suggested to the authors the use of overoxidation and termination as synonyms. Perhaps inspired (although not mentioned) by the encouraging results obtained with potentiostatic pulses, deposition with galvanostatic pulses was examined, and for comparison, simple galvanostatic deposition was included [46]. Different morphologies of the obtained ICPs were observed. The claimed absence of overoxidation certainly expected with these methods was not supported by the presented evidence. Photocatalytic core–shell particles of SiC coated with PANI prepared by photocatalytic synthesis yielded coatings showing no evidence of overoxidation [47].

PANI was electropolymerized on undoped nanodiamond powder without overoxidation when an upper electrode potential limit of *E*_Ag/AgCl_ = 0.9 V was observed during continuous polymerization potential cycling after a few initial cycles to higher values [48]. Oxidative chemical polymerization at the water–chloroform interface with (NH_4_)_2_S_2_O_8_ yielded PPy films of 3–4 μm thickness showing a higher degree of overoxidation (based on electronic conductivity data) at a higher oxidant vs. monomer ratios [49]. The findings were corroborated by results from infrared spectroscopy and elemental analysis. In a calorimetric study of the chemical polymerization of aniline with ammonium peroxydisulfate as an oxidant, evidence of overoxidation in terms of polymer chain hydrolysis and chlorine substitution was found [50]. The expulsion of anions during overoxidation was verified with XPS [51]. Elevated temperatures (*T* > 35 °C) accelerated overoxidation with an attached decrease in electrochemical activity, decreasing conjugation and destruction of the porous polymer structure [52].

Anion (chloride and perchlorate) effects during the electrochemical overoxidation of poly(*o*-methoxyaniline) were studied [53]. A higher solvation of chloride anions (enabling the transport of more solvation water molecules available for subsequent nucleophilic attack into the polymer film) resulted in more degradation than with perchlorate anions.

A coating of poly(2,5-dimethoxyaniline) applied as corrosion protection was found to be more effective, stable, and adhesive than plain PANI, as deduced from its slower oxidation at electrode potentials in the range of electrochemical overoxidation and from the higher inhibition of ion penetration across the polymer film [54].

### 2.2. PPy

**Figure 7:**
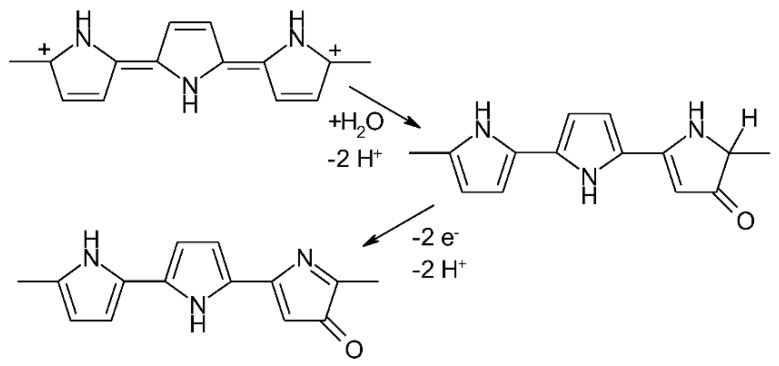
Mechanism of PPy overoxidation starting in the oxidized bipolaronic state (top) via an intermediate yielding PPy with pyrrolinone units interrupting conjugation (based on [63,64]).

The mechanism of PPy overoxidation was explored [55] following much earlier observations of irreversible changes of PPy after exposure to electrode potentials of *E*_SCE_ > 0.8 V in various neutral and acidic electrolyte solutions [56,57]. The formation of hydroxyl radicals, which subsequently chemically attack PPy, has been observed. Accordingly, other modes of overoxidation, e.g., applying Fenton’s reagent [58], can be applied when controlled overoxidation is required in sensor material preparation (see below). In addition, this observation suggests a mode of overoxidation prevention by the addition of radical scavengers. Methanol and dimethylthiourea were suggested [55]. Quinones and quinone oligomers, which were under consideration themselves as active electrode materials for batteries and supercapacitors [59], were suggested as both overoxidation inhibitors [60] and radical scavengers [61]. Using the results of various spectroelectrochemical techniques [62], the mechanism and products of PPy overoxidation in aqueous electrolyte solutions were examined [63]. The formation of pyrrolinones (see Figure 7) with short conjugation lengths and of CO_2_ resulting from the oxidation of terminal units, as well as an influx of solvent into the film (swelling but staying mechanically intact), have been reported.

This study extends the results of a former one wherein the effects of various nucleophiles were examined in detail [64]; the results were confirmed in part later elsewhere [65]. Similar observations with PPy hydrogels, in particular the formation of =O and –OH groups, were reported elsewhere [66]. With XPS, these findings have been confirmed [67,68,69]. Earlier XPS results were in close agreement [70]. PPy was prepared from an electrolyte solution of NaOH at an electrode potential that directly yielded its overoxidized form [71]. XPS data of the films were again in agreement with those mentioned before. Images obtained with scanning tunneling microscopy confirmed the instability of PPy above electrode potentials of *E*_SCE_ < = 0.7 V [72].

Electrochemical degradation of PPy at a rather moderate electrode potential of *E*_SCE_ = 0.58 V was studied with electrochemical impedance measurements and the electrochemical quartz crystal microbalance technique [73]. Apparently, the authors considered overoxidation and degradation as synonyms in this somewhat difficult to understand report. In the impedance data, a large increase in the charge transfer resistance inserted in the equivalent circuit used for data evaluation was noticed. This was taken as an indication of hindered ion insertion. The finally concluded “electrical degradation” was explained in terms of breaking bonds in the polymer chain resulting in carbon dioxide formation and crosslinking. Elsewhere, this is generally called structural or chemical modification. In the reported work, these aspects could not be studied with the applied methods; thus, this conclusion appears to be unsupported. Instead, the authors claimed an “electrochemical degradation”, which they defined as “morphological change in the polymer matrix”. This, in turn, is hard to match with their presented evidence; it is mysterious. The reported instability of PPy against “oxidative conditions” [74] was definitely not called overoxidation, as erroneously claimed in [73]. The first reported use of the term “overoxidation” can be found instead from 1987 in [64,75].

Overoxidation of PPy prepared electrochemically has been studied with in situ infrared spectroscopy using a setup described elsewhere [76,77] in acetonitrile- and propylene-carbonate-based electrolyte solutions [78]. In propylene carbonate, irreversible oxidation yielding carbon dioxide was noticed. With traces of water from the electrolyte solution, hydrogen carbonate and hydroxyl ions as nucleophiles were formed that, in turn, attacked the PPy, leaving a rather inactive polymer. The gradual decrease in the electrochemical activity of PPy in aqueous media corresponded to the increasing degree of irreversible oxidation; upon completing irreversible oxidation, electrochemical activity was completely lost [57]. This oxidation requires about 70% of the charge initially required for polymer formation. Methyl substitution cannot suppress overoxidation. For this purpose, instead, the need for substitution with groups causing higher electron density was concluded. PPy and poly(*N*-methylpyrrole) were prepared by electropolymerization from their aqueous solutions [79]. A PPy film generated with about 180 mC·cm^−2^ needed only about 20 mC·cm^−2^ for electrochemical deactivation by overoxidation; this was at variance with much larger charges (about 70% of the deposition charge) reported elsewhere [57]. The overoxidation of poly(*N*-methylpyrrole) was not examined.

During the stepwise overoxidation of PPy, decreasing effective diffusion coefficients hinting at growing crosslinking connected with lower electronic conductivity and increased polymer rigidity were noticed [80]. These effects slowed down the rate of further overoxidation. Overoxidized PPy showed a high affinity towards dioxygen, and the electrode potential to achieve this sensitivity was lower than the one required to initiate nucleophilic attacks [81]. The electron-withdrawing effect of the attached carbonyl group in poly(3-acylpyrrole) made the polymer backbone more susceptible to nucleophilic attacks; the amount of nucleophilic impurities in the electrolyte solution (e.g., water) could be correlated to the degree of overoxidation [82]. The overoxidation behavior of PPy electropolymerized in either aqueous or organic electrolyte solutions was compared [83]. In the nonaqueous solution, overoxidation proceeded only at much higher electrode potentials without obvious ICP degradation. PPy (although the title suggested a study of several different polypyrroles, only the plain parent polymer was studied) overoxidation in the presence of various weakly solvated boron-containing anions was studied [84]. These ions (for more examples, see below) supported high overoxidation potentials. The higher efficiency of monoionic anions is in agreement with conclusions from an earlier study of the anion-specific effect in overoxidation (see above) [19]. Spatial distribution of anions in the overoxidized PPy layer could be resolved; the highest concentration was found at the film surface. Overoxidation potentials shifted in a positive direction by 300 to 500 mV when using metallacarborane counter anions [85]. A break-in phenomenon was noticed for PPy with polyvinyl sulfonate as a counter anion [86]. A continuous increase in infrared band intensities, as well as in charge under CV peaks, was taken as evidence of a growing number of polymer chains participating in the redox process. At *E*_Ag/AgCl_ = 1.3 V, a band typical of a carbonyl function was found, suggesting the onset of overoxidation. The observed shift of a band around 1540 cm^−1^ to higher wavenumbers indicated a decreasing conjugation length attributed to overoxidation.

The influence of anions on PPy film formation, morphology, and sensitivity towards overoxidation was examined in a comparative study [87]. The reported data did not provide clear evidence regarding the stabilizing and destabilizing effects of anions, which was perhaps similar to considerations discussed above for PANI. In one more comparative study of electrochemical PPy formation with various anions present in the polymerization electrolyte solution, PPy formed in HCl- and KCl-containing solutions were less prone to overoxidation than those formed in a sulfate-containing solution [88]. The highly unusual application of the term “irreversible”, as in irreversible monomer oxidation, leaves some concerns or, at least, confusion. The conceivable effects of solvent composition and anion identity on the overoxidation of PPy were studied [89]. The redox activity of PPy in the presence of halide ions has been monitored in the UV-Vis range, and changes in the UV-Vis spectra have been interpreted in terms of different charge carriers and changing molecular structures [90,91]. In the presence of fluoride anions, higher oxidation states, possibly at the expense of overoxidation, were observed. Further details regarding this aspect, particularly kinetic minutiae, were reported [92]. The particular effect of fluoride anions compared to nitrate and chloride, particularly the overoxidation at less positive electrode potentials, was attributed to nucleophilic attacks by hydroxyl ions generated by water dissociation [93]. This reasoning seems to lack coherence because the same solvent has been used with the other anions. More likely, an anion-specific effect (charge density, ion size, or shielding efficiency) as already addressed above was at work. PPy prepared electrochemically from aqueous solutions with either tetrafluoroborate or *p*-toluenesulfonate as counter anions have been studied with pyrolysis mass spectrometry supported with infrared spectroscopy [94,95]. High concentrations of oxygen defects, in particular, after deposition at higher electrode potentials and thermal aging were noticed. PPy prepared electrochemically in an electrolyte solution of *p*-toluenesulfonate did not show overoxidation [96].

PPy was prepared by chemical oxidation supported by UV radiation [97]. The fraction of obtained overoxidized PPy depended on the concentrations of H_2_O_2_ and H_2_SO_4_, with growing concentrations of acid resulting in less overoxidation and higher electronic conductivity.

The controlled electrochemical overoxidation of PPy resulted in anodic voltammetric charge losses associated with shorter linear chain segments within the stimulated conformational relaxation model [98]. Caloric effects and structural changes of PPy during electrochemical overoxidation were studied [92]. PPy electrodeposited on austenitic steel showed a major loss of optical absorption around 780 nm (bipolaron region) when overoxidized in a neutral nitrate electrolyte solution [99].

In a study of PPy electropolymerized on the aluminum alloy 6061-T6 as an interlayer by potentiodynamic and potentiostatic procedures, the optimum deposition potential and upper potential limit were determined, but films obtained with both procedures provided no corrosion protection [100]. PPy was deposited potentiodynamically and galvanostatically from aqueous solutions with tartaric acid as a supporting electrolyte [101]. According to the results of infrared spectroscopy, galvanostatic depositions at relatively higher current densities were more prone to overoxidation. Films prepared at low current densities performed best in corrosion protection. Thin films of PPy electropolymerized on mild steel for corrosion protection were substantially overoxidized according to XPS findings [102].

In a template-free direct electrochemical formation of PPy arrays, the obtained PPy wires were coated with overoxidized PPy [103]. From an alkaline electrolyte solution, micro- and nanosnails of PPy and mostly overoxidized layers of PPy could be formed [104]. The influence of anions and solution pH on PPy micro- and nanostructures obtained preferably without a template has been discussed [105,106], and possible overoxidation depending on the particular experimental conditions and actual interfacial situation was addressed. The formation of PPy microcontainer and doughnut structures prepared via various electrochemical procedures with different anions was attributed to the overoxidation of fluoride-containing polymer [107]. Unintended overoxidation possibly resulting in PPy with lower electronic conductivity was examined as a conceivable reason for the existence of maximum conductivity [108]. The overoxidation of PPy caused changes in impedance measurement results, in particular, of electronic conductance and diffusion coefficients [109].

Galvanostatically (0.1 < *j* < 2 mA·cm^−2^) prepared free-standing PPy films with *p*- toluene sulfonate as a counter anion showed better mechanical and electronic properties in electrochemomechanical deformation studies when formed at lower current densities [110]. At the employed current densities, the obtained films did not show signs of much overoxidation based on the carbonyl band in the infrared spectra of the films. The galvanostatic formation of nanometer-thick films of PPy on Au(111) from solutions with low pyrrole concentrations examined with STM and several spectroscopies verified the possibility of preparing overoxidized films directly [111]. Direct current and pulsed direct current (i.e., galvanostatic) depositions of PPy films for use in supercapacitor electrodes were compared [112]. Films obtained with the former method showing carbonyl stretching mode bands in infrared spectra were taken as evidence of overoxidation, but PPy obtained with the second method were not tested. The electronic conductivity of the latter material was higher by two orders of magnitude. The larger effective diffusion coefficient suggesting a higher porosity enabled a better high-current performance of this material that was stable for a much longer time.

**Figure 8:**
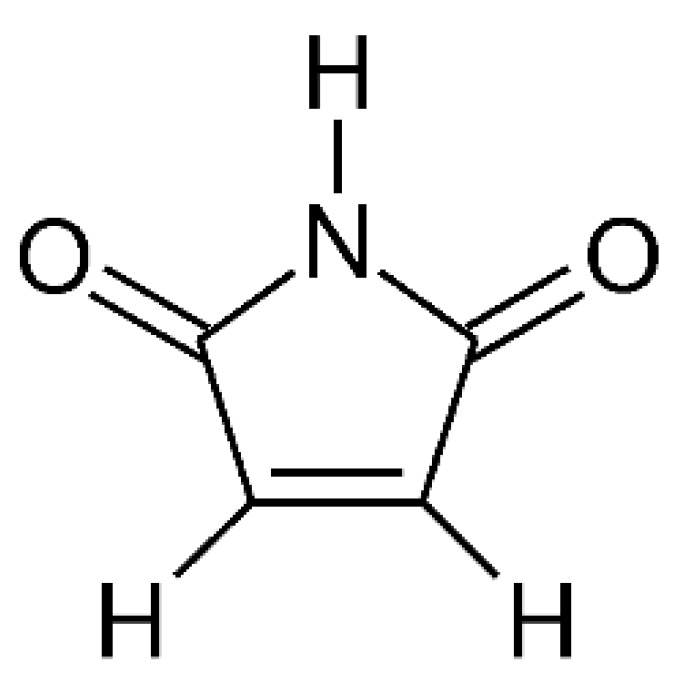
Maleimide.

Overoxidation, particularly the formation of associated defects, during electropolymerization could be avoided by using bipyrrole or substituted bipyrroles requiring lower oxidation potentials as starting monomers [113]. During chemical polymerization with FeCl_3_, conceivable overoxidation evidenced by electronic conductivity [114] could be reduced by adding FeCl_2_ [115]; this is essentially equivalent to a lower electrode potential in electro-oxidation. As expected, the rate of formation decreased. Using HPLC, maleimide (Figure 8) was identified as an overoxidation product.

**Figure 9:**
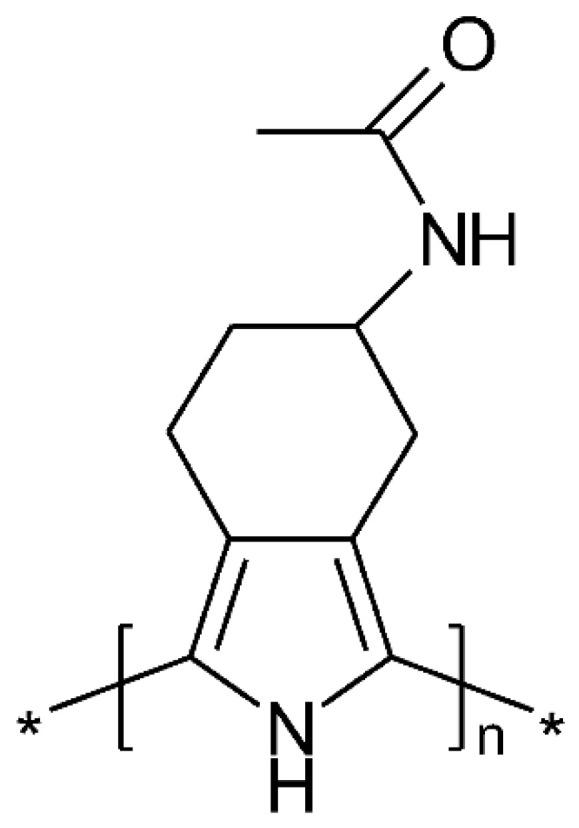
Poly(5-acetamido-4,5,6,7-tetrahydro-2H-benzo[c]pyrrole).

Suitably substituted 5-acetamido-4,5,6,7-tetrahydro-2H-benzo[c]pyrrole (Figure 9) has been polymerized chemically into an ICP showing considerably larger storage capability (0.8 e per repeat unit, instead of 0.3 without such a substituent) without loss of electroactivity due to overoxidation [116,117]. Stabilization of the polymer has been attributed to the polar substituent.

**Figure 10:**
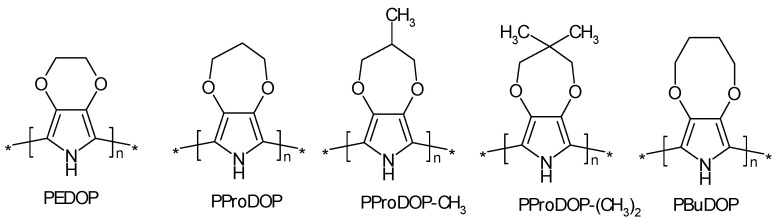
Poly(3,4-alkylenedioxypyrrole)s.

ICPs prepared by the electropolymerization of several dihydro-benzodipyrroles underwent easy overoxidation (in terms of low oxidation potentials) without losing their charge storage capability [118]. This was also observed with several 3-alkylthiopyrroles [119]. Several poly(3,4-alkylenedioxypyrrole)s (Figure 10) and their electrochemically prepared ICPs were synthesized and compared; their overoxidation started at about 2 V positive to the redox potentials of these polymers [120].

Nanoparticles of PPy were obtained by chemical polymerization with various oxidants; longer polymerization times resulted in more overoxidation, evidenced with lower electronic conductivity [121]. PPy nanoparticles prepared by chemical polymerization as platinum support for ethanol oxidation electrodes prepared in a microemulsion system did not show evidence of overoxidation [122]. Powdery PPy was obtained electrochemically with an aluminum electrode and an aqueous HNO_3_ electrolyte solution [123]. No overoxidation (as frequently observed with other electrode materials) was noticed.

The overoxidation of PPy in aqueous electrolyte solutions containing perchlorate or *p*-toluenesulfonate was less severe in the presence of the organic anion. An explanation possibly invoking differences between the anions and the polymer chain (as suggested above in studies of anion effects in the overoxidation of PANI) was not provided [124].

Permselectivity, as a particularly intriguing property of overoxidized PPy (elsewhere called TPP-treated polypyrrole, OPPY, or OPPy-overoxidized PPy), has been studied repeatedly [125,126,127,128]. The substrates (glassy carbon and rough pyrolytic graphite) significantly influenced properties of overoxidized PPy films [129]. The properties of highly porous PPy membranes and dense films of PPy were compared [130], and results regarding the overoxidation examined also were inconclusive but indicated that properties of the porous membranes were affected less. Ultra-thin overoxidized electrically insulating, pin-hole free PPy films were prepared in a one-step process [131]. XPS confirmed the absence of counter anions in such very thin overoxidized PPy films [132]. Protections against electrode fouling by surfactant and protein adsorption were noticed [133]. Photocurrents measured with PPy decreased upon overoxidation [134]. Chemical overoxidation and, finally, material degradation by 8 **M** nitric acid was observed [135]. The influences of various pretreatments of PPy (including overoxidation) on ion sensitivity were examined with inconclusive results [136]. Further analytical applications of the permselectivity of PPy are discussed in a following chapter.

The onset of overoxidation of PPy and its copolymer with *N*-methylpyrrole prepared in nonaqueous electrolyte solutions was examined with electrochemical and spectroscopic tools [137]. No influence of copolymer composition on the onset potential was noticed; obviously, copolymerization does not help in expanding the useful electrode potential to more positive limits. In a comparison of PPy and poly(*N*-methylpyrrole), it was noted that the redox potential of the latter polymer was higher by about 0.6 V, whereas overoxidation became noticeable for both polymers at about the same electrode potential [138]. Accordingly, the useful storage capability of PPy was larger. The permeability of poly(*N*-methylpyrrole) for protons depended on the counter anion: with nitrate, the film was closed, but with tosylate, it was permeable [139].

Poly(*N*-methylpyrrole) electropolymerized on copper as a corrosion protection turned out to be in its overoxidized state after preparation, and corrosion protection was attributed to a barrier effect of this coating [140]. The corrosion protection performance of PPy coatings on stainless steel as a function of the electropolymerization potential was studied [141]. Coatings deposited at lower potentials were more homogeneous, showed (not unexpectedly) less overoxidation, and provided better protection.

**Figure 11:**
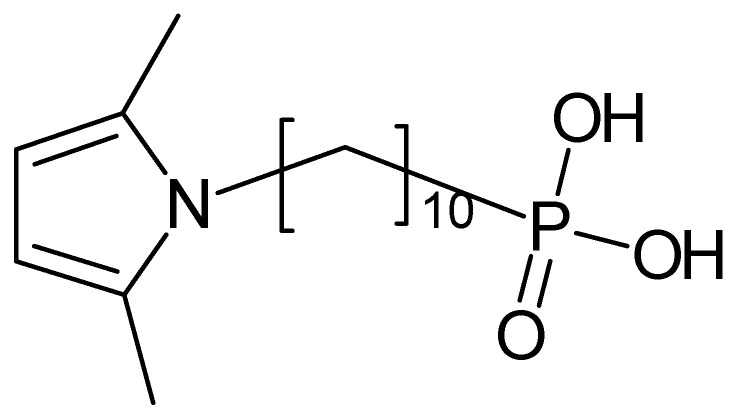
Repeat unit of poly(1-pyrrolyl-10-decanephosphonic acid).

Poly(1-pyrrolyl-10-decanephosphonic acid) (Figure 11) was electropolymerized, and crosslinking was suggested as an effect of overoxidation [142].

**Figure 12:**
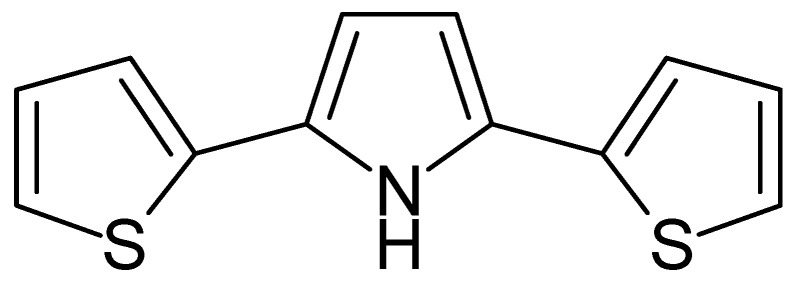
2,5-Di(-2-thienyl)-pyrrole.

Poly[2,5-di(-2-thienyl)-pyrrole] (Figure 12) was electropolymerized from a nonaqueous electrolyte solution and studied in its reduced (presumably, neutral state is meant), oxidized, and overoxidized states in a water and acetonitrile medium [143]. The overoxidized state was electroinactive, meaning counter anions could not be expelled from it.

In studies on a composite of PPy and WO_3_ considered as an electrochromic material, a degree of overoxidation *Y* depending on the nucleophile present in the electrolyte solution based on the charge *Q*_oo_ was observed in a CV associated with overoxidation and the reversible redox charge *Q*_red_ according to:

$$Y=\frac{Q_{\mathrm{oo}}}{Q_{\mathrm{red}}}$$

where a value of *Y* = 5 was found when water was present [144]. The metal oxide apparently did not influence the overoxidation. Composites of clay and PPy were formed by the spontaneous oxidation of pyrrole monomers by ferric sites in, for example, montmorillonite [145]. In situ FTIR-ATR spectroscopy did not show any signs of overoxidation products. Whether the limited mass transport suggested by the authors was the cause remains uncertain.

Connections between overoxidation and self-discharge were discussed for PPy electrodes in terms of a reaction intermediate appearing in a “polymer intrinsic endergonic electron transfer reaction” [146]; an overview on discharge in electrochemical energy storage, particularly supercapacitors, is available [147]. Overoxidation of the positive electrode in an all-PPy battery with different redox-active counter ions in the electrodes was the major reason for rapid capacity decay (50% after 60 cycles) at rather low current densities [148].

### 2.3. PTh

In a spectroelectrochemical study of the overoxidation of PTh, UV-vis spectroscopy revealed an almost complete loss of optical absorption attributed to π→π*, the nucleophilic substitution by electrolyte anions was confirmed, and oxygenated products were identified in addition [149]. Changes of physicochemical properties and corrosion protection performance of PTh films were examined [150]. Structural defects resulting in shorter conjugation length, lower electrochemical activity, and conduction were observed. Corrosion protection was negatively affected by poorer adhesion and degraded mechanical properties. The corrosion of various polythiophenes has been studied [16,17]. Two processes, an electrochemical and a chemical (nucleophilic attack) one, could be distinguished. Different from the behavior of PPy, reversible oxidation of sulfur atoms was found without interruption of the conjugation of the polymer backbone. Three polythiophenes were studied in detail in the presence of various amounts of water; the nucleophilic capability of water during overoxidation was of particular interest [151]. Overoxidation electrode potentials and redox potentials of reversible oxidation processes were close, and partial overoxidation had no effect on electronic conductivity. At large water concentrations, oxidative SO_2_ elimination and formation of carbonyl groups with associated further structural changes proceeded. In a study on PTh formation, evidence for a competition between oxidative electropolymerization and overoxidation was observed [152], which confirmed earlier observations [153]. The latter authors coined the term “polythiophene paradoxon”, describing when PTh is overoxidized at electrode potentials required for its formation by electropolymerization. As a consequence, the latter authors assumed that PTh may actually be a composite or even a copolymer of PTh and its overoxidation products.

As part of a composite with carbon and a sonogel, alkylsulphanyl-substituted polythiophenes were more stable against overoxidation compared with the plain polymer [154]. Impedance measurement results for PTh at various states of oxidation were reported [155]. In the range of overoxidation, a steep increase in the Ohmic film resistance was observed.

An increase in the charge transfer resistance as a function of the duration of overoxidation was also found during the overoxidation of poly(3-hexylthiophene) [156]. The decrease in double layer capacity suggested a loss of electrochemically active surface area indicative of structural changes and loss of material. The decrease in the capacitance associated with redox storage supported this assumption. Poly(3-dodecylthiophene) prepared both galvanostatically and by CV was studied with an EQCM [157]. In the case of slow monomer diffusion, overoxidation of the formed polymer could occur. Copolymerization of 3-methylthiophene and thiophene-3-acetic acid did not show evidence of a Kolbe reaction at electrode potentials where overoxidation was expected [158]. The results suggested that polymerization started with the radical formation of 3-methylthiophene, which was oxidized at lower electrode potentials than the comonomer, and overoxidation of the copolymer proceeded at much higher electrode potentials, leaving a wide potential range for reversible charge storage.

The electronic band structure of polybithiophene was probed using various redox systems in an electrolyte solution [159]. Electronic states (mini bands) were detected in the electronic bandgap, and evidence suggesting band structure changes during overoxidation was offered. Polybithiophene deposited photoelectrochemically on *n*-doped silicon did not show evidence of overoxidation, and the striking mismatch between anodic and cathodic charge in optical switching (5:1) was explained by assuming side reactions on the silicon surface [160]. Doping levels (defined as the amount of charge injected taking into account side reactions) were determined for polybithophene and poly(3-methylthiophen), and the applied procedure enabled the separation of charge injection and overoxidation [161]. Polybithiophene with poly(β-hydroxyether) as a counter anion showed a relatively high electrode onset potential for overoxidation [162].

**Figure 13:**
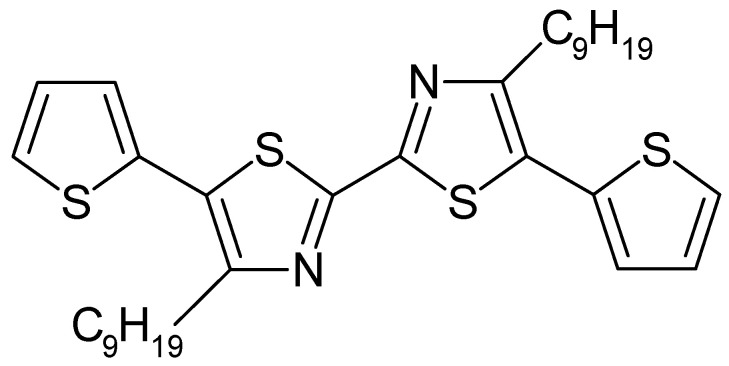
Bis(thiophene)-(4,40-dinonyl-2,20-bithiazole).

Bis(thiophene)-(4,40-dinonyl-2,20-bithiazole) (Figure 13) was electropolymerized into a film on a carbon fiber electrode [163]. The claimed stability against overoxidation remains a claim lacking comparative evidence.

Poly(3-methylthiophene) prepared galvanostatically in a nitrobenzene-based electrolyte solution was examined in a wide electrode potential range without overoxidation in an attempt to resolve the long discussion about the separation of Faradaic and capacitive currents [164]. The characterization of the electronic and ionic properties of poly(3-methylthiophene) yielded significant differences between charges under anodic and cathodic peaks in CVs attributed to overoxidation [165]. Mild electrochemical overoxidation of poly(3-hexylthiophene) resulted in a loss of redox capacity and an increase in charge transfer resistance attributed to unspecified interfacial Faradaic processes [156]. In an earlier report by these authors, the aging of this polymer (presumably, poly(3-hexiltiophene) is poly-3-hexylthiophene) was afforded by applying an overoxidation potential [166]. Using the bending beam method, the shrinking of a film of poly(3-octylthiophene) during overoxidation was recorded [167]. Loss of counter anions, destruction, and delamination were suggested as possible causes of the shrinking. Thiophenes substituted with various mesogenic group spacers of different chain lengths were electropolymerized, and the competition between overoxidation and polymerization proceedings at more- or less-closely spaced electrode potentials was observed again [168].

### 2.4. PEDOT

Although EDOT is a substituted thiophene and should have been included in the previous section, the large number of reports dealing with it and its large popularity justify a separate section. In comparative studies of PPy, PEDOT, and their copolymers electrochemically synthesized in a nonaqueous electrolyte solution, the large potential difference between deposition and overoxidation electrode potential for PEDOT has been highlighted [169,170]. From an aqueous electrolyte solution, PEDOT (rarely also called PEDT) was formed by electropolymerization in the presence of surfactants, increasing the solubility of EDOT [171]. Evidence of overoxidation at too-high electrode potentials was obtained with UV-vis spectroscopy. The electropolymerization of EDOT in an aqueous solution without added surfactant was monitored with various methods [172]. Evidence of overoxidation after longer polymerization times was collected with UV-vis spectroscopy, in particular, when too-high electrode potentials were applied. The influence of electropolymerization potential on the properties and behavior of PEDOT prepared in an aqueous electrolyte solution without surfactants was studied [173]. In a CV recorded with the monomer, two current peaks were found around 0.8 and 1.4 V vs. an SCE. With the PEDOT coating, only the latter peak was observed, which decreased rapidly in the subsequent CVs, suggesting fast deactivation of the ICP. Unfortunately, only the first cycle was displayed, and because potentiostatic electropolymerization proceeded at significantly lower electrode potentials, this peak in the first scan was only needed for initiation of the polymerization. The author’s claim that polymerization and overoxidation potential were very close remains misleading at best. Highly conducting PEDOT could be electropolymerized from aqueous solutions with about the same conductance as PEDOT obtained from nonaqueous solutions [174]. Infrared spectra did not reveal ring opening or other evidence of overoxidation.

Electrochemical degradation, including the overoxidation of PEDOT, has been studied and reviewed [175,176,177], providing more details to the rather general statement of polymer degradation upon the overoxidation of PEDOT in aqueous electrolyte solutions [178]. Structural changes resulting in mechanical stress, the cracking of polymer layers, the delamination of coatings, and the release of soluble degradation products were discussed. After electrode potential excursions in CVs slightly above *E*_SCE_ = 0.8 V, some overoxidation was claimed to occur with changes barely visible in the CVs, but only after applying an upper limit of *E*_SCE_ = 1.5 V were significant losses of redox capacity observed. Impedance measurements suggested, in addition, a decrease in electrochemical activity noted as an increased charge transfer resistance. Suggested overoxidation mechanisms for PTh including nucleophilic substitution at the thiophene ring with further chemical changes and ring cleavage were discussed. Although the ethylene-dioxy group substitution at the thiophene ring in PEDOT basically prevented some of these reactions, the overoxidation of PEDOT was claimed to be essentially the same as that of PTh. Instationary changes of charge transfer resistance during overoxidation and immediately thereafter have been examined with electrochemical impedance measurements [179,180]. The observed behavior suggests a certain “healing behavior”. Changes of PEDOT films upon overoxidation in aqueous sulfuric and sulfate electrolyte solutions have been monitored with the bending beam method [181,182]. Beyond confirmation of these findings, in one more report by these authors the X-ray diffraction of overoxidized PEDOT suggested increased crystallinity because of the decreasing widths of more intense scattering peaks [180,183]. The influences of electrode potential time protocols and the duration of electrochemical overoxidation have been examined in detail; morphological differences visible microscopically and different performance in lead ion determination were reported [184,185].

Large volume and height changes possibly useful in actuator applications were reported for PEDOT:PSS within the electrode potential regime of reversible redox reactions. Outside of the reversible operating regime (which is different from the common electrochemical arrangement for this actuator), irreversible overoxidation of the ICP proceeded [186].

**Figure 14:**
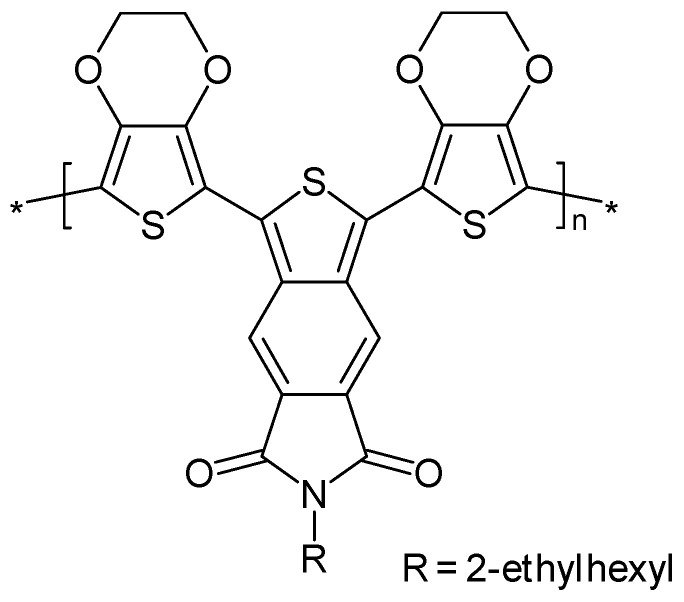
Poly(1,3-bis(2′-[3′,4′-ethylenedioxy]thienyl)-benzo[c]thiophene-*N*-2″-ethylhexyl-4,5-dicarboximide).

Electropolymerized poly(1,3-bis(2′-[3′,4′-ethylenedioxy]thienyl)-benzo[c]thiophene*N*-2″-ethylhexyl-4,5-dicarboximide) (Figure 14) was identified as a stable, low-band-gap ICP with high charge storage capability that was very stable against overoxidation; it could be both reduced and oxidized [187]. The low oxidation electrode potential was easily linked to extended conjugation in the monomer.

The overoxidation of PEDOT results in higher crystallinity. This was attributed to the formation of smaller oligomer units during overoxidation [188]. In the case of a perchlorate-selective sensor, the overoxidation of PEDOT did not result in improved stability (as frequently observed elsewhere), possibly because of the already high stability of this ICP compared with, for example, PPy [189].

PEDOT electropolymerized in solutions containing various supporting electrolytes, including anionic polysaccharides, was more stable vs. overoxidation and appeared smoother and denser than polymers formed in the presence of other electrolytes [190].

PEDOT can also be formed by oxidative jet deposition, enabling inline processing less easily performed with common oxidative batch processes (chemical and electrochemical oxidation) [191]. With properly adjusted conditions, plasma jet deposition yielded less overoxidation than ozone jet deposition.

**Figure 15:**
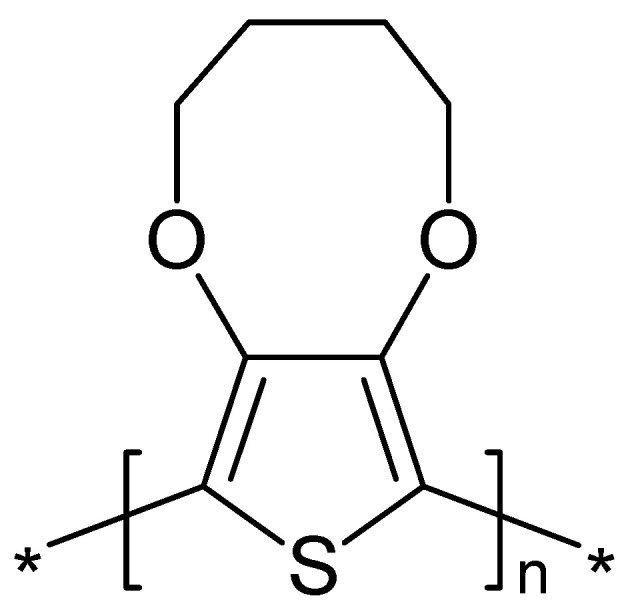
Poly(3,4-butylenedioxythiophene).

The effect of the electrochemical overoxidation of poly(3,4-butylenedioxythiophene) (Figure 15) on charge carriers (polarons) was studied with electron paramagnetic resonance spectroscopy [192]. Earlier findings with plain PEDOT by the same authors were confirmed [193].

Observed irreversible changes in the EPR spectra, particularly the steep drop of spin density, were attributed to overall degradation of the polymer and, in particular, to a decrease in the conjugation length by crosslinking or addition reactions with remaining spins confined to isolated segments of the polymer chain.

Polymers formed by the electropolymerization of tetrathiafulvalene-substituted EDOT showed reduced electroactivity of the substituents after overoxidation, which was attributed to diminished activity of the polymer backbone [194].

### 2.5. Miscellaneous ICPs

Bilayer systems of different ICPs were studied aiming at diode-like behavior [195]. Detrimental effects of overoxidation were observed when a second layer had to be deposited at electrode potentials where the underlying first layer already faced overoxidation. Similar observations were reported for further bilayer systems [196].

Infrared spectroscopy of overoxidized polyphenylene yielded evidence of a breakdown of the polymer into shorter segments, and para-quinoid sequences were apparently destroyed [197].

A composite of polypyrrole–polyoxyphenylene was electrosynthesized at a relatively high electrode potential needed for oxidation of the second component, 2-allylphenol [198]. Because of the relatively high deposition potential, the obtained composite already had a fraction of overoxidized PPy, and deposition at accordingly lower potentials failed.

A composite of poly(3-methylthiophene) and overoxidized PPy was prepared by applying a specifically designed electrode potential time program and monomer concentrations [199]. The average band gap energy of the composite was larger than that of former homopolymers.

Within studies of the electrochemistry of ionically functionalized polyacetylene analogues, evidence of overoxidation was found [200].

**Figure 16:**
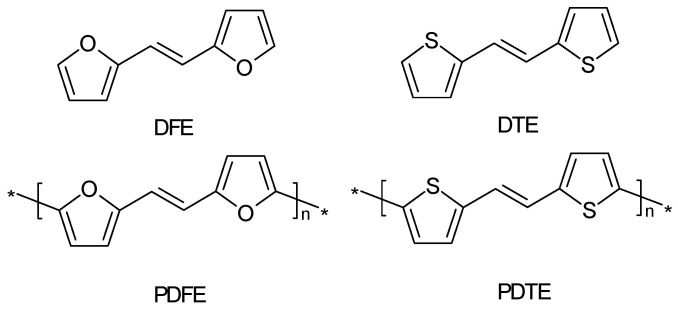
Poly[trans-1,2-di(2-furyl)ethylene] and poly[trans-1,2-di(2-thienyl)ethylene].

The sensitivity towards overoxidation of poly[trans-1,2-di(2-furyl)ethylene] DFE and poly[trans-1,2-di(2-thienyl)ethylene] DTE (Figure 16) was compared [201]. According to CVs of the polymer films, the former was overoxidized more easily, and its electronic conductivity was lower by several orders of magnitude.

The differences in material properties between PDFE and PDTE must be primarily related to differences between sulfur in the thiophene ring and oxygen in the furan ring, although this is nowhere addressed in the report. Oxidation of the monomers showed a behavior hardly helpful in finding an explanation: DFE was oxidized at substantially lower potentials than DTE, as seen in the presented CVs.

**Figure 17:**
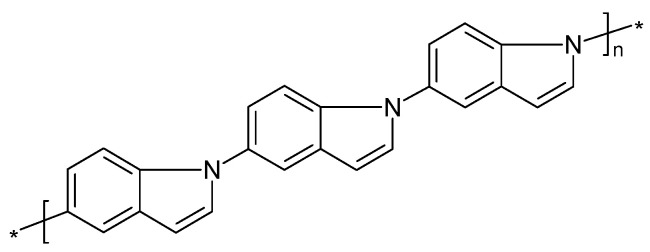
Polyindole.

The second oxidation step in a CV of polyindole (Figure 17) was associated with overoxidation [202].

In a report on the preparation of nanosized polyindole by emulsion polymerization, overoxidation was addressed only as a keyword [203]. The permselectivity of overoxidized polyindole was studied, and generated carboxylate functionalities and cation permselectivity were noticed [204].

**Figure 18:**
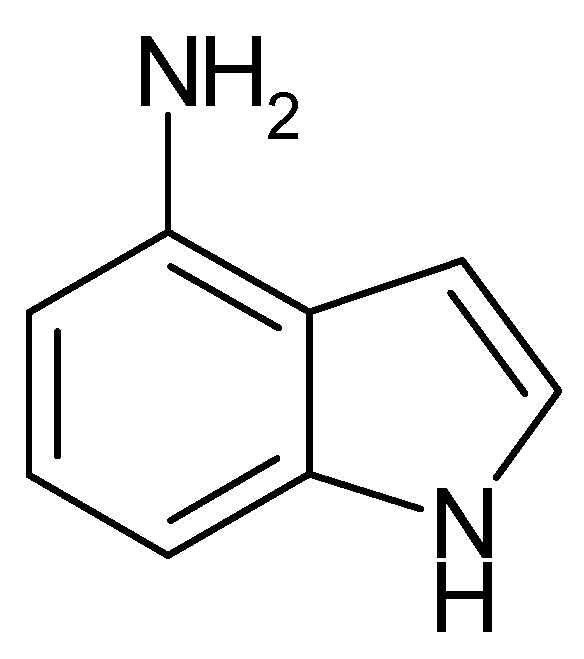
4-Aminoindole.

The adsorption and polymer film formation of 4-aminoindole (Figure 18) on platinum and gold electrodes were studied with the electrochemical quartz crystal nanobalance method [205].

With a platinum electrode, already dioxygen was sufficient to start oxidative adsorption and deposition of a multilayer film on the electrode surface. Electrochemical oxidation of the monomer also resulted in polymer film formation on both metals with film properties depending on the applied electrode potential. Overoxidation may proceed both by dioxygen and high electrode potentials. It appears noteworthy to notice that linkage between monomer units proceeds only via the pyrrole entity leaving the amino group at the benzene ring available for functionalization. At too-high electrode potentials, this group may be oxidized, too, resulting in a loss in electroactivity of the film.

The overoxidation of polymers of bis(salen) complexes resulted in morphology changes affecting effective diffusion coefficients and crosslinking [206].

Poly[(tetraethyldisilanylene)oligo(2,5-thienylene)] derivatives showed overoxidation of the oligo(thienylene) units and Si–Si bond cleavage upon exposure to too-high electrode potentials, resulting in lower conductivity and values of the work function [207].

**Figure 19:**
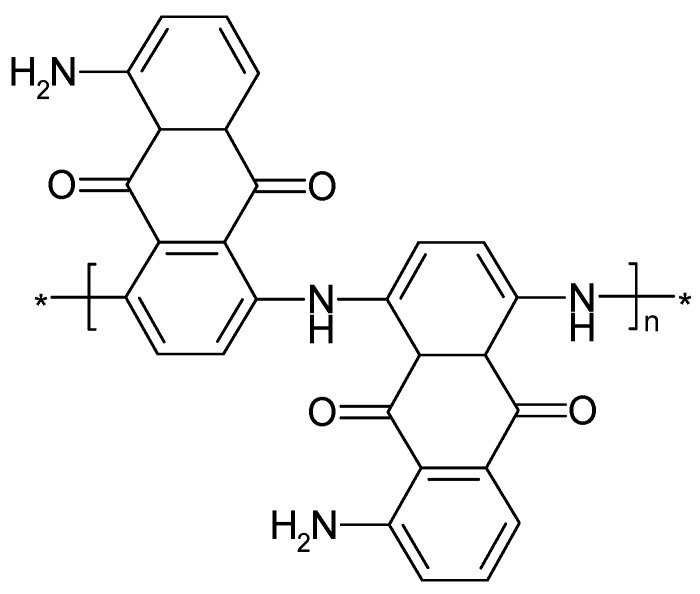
Poly(1,5-diaminoanthraquinone).

Poly(1,5-diaminoanthraquinone) (Figure 19) can be prepared by electropolymerization. Among various uses, its applications in electrochemical energy storage and conversion utilizing the redox capability have been reported [59,208]. Further results obtained with oligomeric material are available [22].

Degradation of this polymer during electrode potential cycling has been observed both during overoxidation (when the electrode potential exceeded the range of the quinone–hydroquinone redox process) and the basically reversible redox reaction, with a much lower rate in the latter case. During overoxidation, the quinone structure is destroyed, the conjugation length decreases, the polymer chain may be broken, and chemical follow-up reactions may occur. In case of oligomeric material showing π-π stacking, conformational changes may play a role, too [208].

### 2.6. How to Inhibit, Prevent, or Meliorate Overoxidation

At first glance, avoiding electrochemical overoxidation appears to be straightforward and simple: set a proper upper (positive) electrode potential limit. This might work in an experimental lab setup with a three-electrode arrangement. However, during potentiodynamic deposition of an ICP, overoxidation may already happen, e.g., during the initial scans where, for deposition of at least some ICPs (e.g., PANI), higher electrode potentials are needed to overcome nucleation inhibition or a very poorly conducting polymer film. In the cases of galvanostatic or current pulse deposition without real electrode potential control, the situation is even worse. Frequently, CVs of PANI prepared potentiodynamically show the middle-potential redox peak pair discussed above with the newly formed CP already. In a two-electrode arrangement, as encountered in a secondary battery [25] or a supercapacitor [59,209,210], electrode potential control is impossible. Certainly, the cell voltage can be limited, and by following the approach successfully employed in NiCd batteries for decades (antipolar mass [1]), the proper adjustment of positive and negative mass (with a positive mass slightly larger than actually needed) probability of overcharge and overoxidation may be reduced. Nevertheless, further options have been studied and should be explored more deeply when considering the application of ICPs in supercapacitors.

Considerations as outlined above for PANI also apply to PPy, its synthesis, and its handling. As reported, even at low current densities and in very dry electrolyte solutions, overoxidation was observed during galvanostatic electropolymerization [211]. With self-doped PPy with a cobalt bisdicarbollide moiety attached as a counter anion by a diether aliphatic spacer to the PPy chain, no current peak attributed to overoxidation was observed up to *E* = 1.8 V. Simple PPy showed a strong peak under the same conditions already around *E* = 0.9 V [212]. The low charge density of this anion was invoked as one reason for the stability. Even without covalent attachment and self-doping, this counter anion afforded significant overoxidation stability [213]. Reduced concentrations of salicylate anions widened the window of electrochemical stability versus overoxidation of PPy in an application as corrosion protection coating [214]. Even though the reasons for this enhanced stability without a loss in activity have not been explored, it appears interesting to invoke the same reason already discussed with the previous examples of larger anions and their effects. Another general suggestion to avoid the degradation of an ICP by nucleophilic attacks on cationic sites in the oxidized form of the ICP was simply the use of solvents and electrolytes showing low nucleophilicity; in particular, water should be avoided when possible [215]. This straightforward approach was extended in a wider study of options to improve the stability of positive battery electrodes by using nonaqueous electrolyte solutions [216]. According to observations reported elsewhere, traces of water in the electropolymerization solution have appeared to be helpful in generating a regular growth of more conjugated PPy with small amounts of chains terminated with fragments from the electrolyte solution solvent [210,217].

Using the Al^3+^ cations in Al(NO_3_)_3_ as electrolytes instead of HCl resulted in improved the cycling stability of PANI in a supercapacitor application [218]. The middle-potential peak in the CVs (see above) was less pronounced, confirming the assumed higher sensitivity of PANI to degradation in acidic electrolyte solutions resulting in the already discussed degradation products.

Using oligomers instead of monomers as starting materials is an option to keep the upper electrode limit during electropolymerization at more moderate levels because, generally, oligomers (e.g., bithiophene or terthiophene, instead of thiophene) are oxidized at lower electrode potentials [219].

PPy nanofibers electrodeposited in a two-step process turned out to be more overoxidation-resistant. Different counter anions in the core and the outer layer suppressed removal of the anions from the core, which kept the core electronically conductive and hindered overoxidation of the shell [220].

Use of a “dopant-phobic” electrolyte solution prevented the release of dopant anions from PPy, thus avoiding overoxidation [221]. A hydrophobic nature and the electron-withdrawing character of the attached carborane cage have been suggested as reasons for the resistance of polymers, starting with 3-(neutral ortho- and anionic nido-carborane cage)-substituted pyrroles against high positive electrode potentials [222,223,224]. A composite of PPy and polyethylene glycol containing 5% of the latter was apparently less sensitive to overoxidation by dioxygen from air, possibly by limiting its access to the ICP [225].

**Figure 20:**
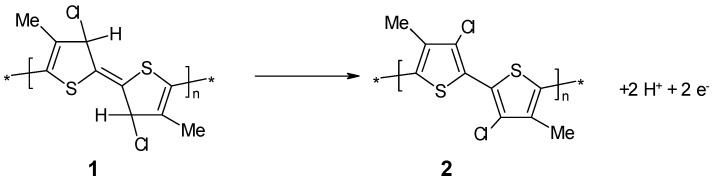
Electrochemical reactivation of overoxidized poly(3-methylthiophene).

Poly(3-methylthiophene) electrochemically overoxidized (**1**) in the presence of chloride ions could be reactivated electrochemically, (**2**) as well as chemically, by hydrogen elimination, as illustrated in Figure 20 [226].

PTh with *meso*-tetraphenylporphyrin (with various metal ions) attached to the ICP backbone via an electronically insulating alkoxy chain was prepared, characterized, and suggested as active material for the detection of polychlorinated phenols in amperometric sensors [227]. Redox features of the PTh and the attached substituent were observed as well-separated because of the insulating bridge. The high stability versus overoxidation (evidenced by an upper potential limit in CVs resulting in material degradation much higher (0.4 .. 0.5 V) than observed with plain PTh) was attributed to delocalized polaronic and bipolaronic states of the ICP combined with charge hopping via the substituents, resulting in partial charge transfer from the ICP chain to the substituents.

PEDOT with poly(3-methyl-2-{[3-(4-vinyl-benzyl)-3H-benzothiazol-2-ylidene]-hyd-razono}-2,3-dihydro-benzothiazole-6-sulfonic) acid as a counter ion turned out to be particularly overoxidation-resistant [228]. This was attributed to the non-leachable counter ion, which in addition provided a third electrochromic state.

The electrochemical overoxidation of polyacetylene could be suppressed by various additions, which were either oxidized themselves at too-high electrode potentials or helped to establish an equal state of polyacetylene oxidation [75].

## 3. Application Possibilities of Overoxidation

Contrary to the mostly negative effects of overoxidation, there are also reports on positive ones. They range from merely increased stability of a particular function of an ICP to the achievement of further capabilities of an ICP. Examples range widely across fundamental and applied electrochemistry, sensors, etc. In the following, they are briefly presented, and particular attention is paid to details revealing correlations between these changes caused by overoxidation and the obtained improvements and effects. It is noteworthy that the overoxidation of a plain carbon microfiber enhances dopamine adsorption and, consequently, sensitivity in the detection of this substance at the expense of a slower response that is possibly related to the significantly increased double layer capacitance [229]. This approach apparently does not afford the welcomed selectivity provided by coatings with ICPs. The stability of PPy and overoxidized PPy films, including “repulsion conditions from the surface” on gold electrodes, was examined [230].

Flexible overoxidized PPy films were examined as negative electrode materials for sodium- and lithium-ion batteries with promising results [231].

A Pt–Co fuel cell catalyst supported on PPy carbon multiwall nanotubes employed in a direct methanol fuel cell showed enhanced catalytic activity after partial overoxidation [232]. PPy with phosphopolyoxomolybdate P_2_Mo_18_$O_{62}^{6-}$ as a counter anion was examined as an electrocatalyst for bromate ion reduction showing activity without and with the PPy being overoxidized [233]. A reduction of chlorate was only slightly catalyzed; this showed a way to detect bromate in water. Overoxidized PPy modified with cobalt tetrasulphonated phthalocyanines showed catalytic activity towards the electrooxidation of 2-mercaptoethanol [234].

Overoxidized PPy as part of a composite also containing cellulose was examined as a separator for a lithium metal battery [235]. The well-defined pore structure of the composite enabled a separator preventing short-circuits.

Favorable changes of the PPy layer coating on a copper support with embedded NiO_x_ nanoparticles showed morphological changes after overoxidation, enhancing methanol transport in the methanol oxidation reaction of a fuel cell electrode [236].

The electrodeposition of atomic gold on PANI was complicated by polymer degradation during the procedure [237]. “Stabilization” of the PANI by overoxidation was found to be helpful with keeping the imine coordination sites for chloroaurate anions present. Cobalt oxide nanoclusters embedded in overoxidized PPy were used for a nonenzymatic glucose sensor, showing high electrocatalytic activity and good anti-interference capability that was attributed to the overoxidation [238]. A fast amperometric nonenzymatic hydrogen peroxide sensor based on overoxidized PPy containing palladium was tested [239]. High selectivity for serotonin of overoxidized PPy with dodecyl sulfonate micelles as counter anions for an amperometric sensor was attributed to the high negative charge carried by the anion micelles [240]. An amperometric microbiosensor for L-glutamate based on platinum black deposited on a platinum microdisc coated with permselective overoxidized PPy and a top layer of L-glutamate oxidase crosslinked with glutaraldehyde was reported [241]. Selectivity vs. dopamine, fast response time, and sufficient stability for applications (including in vivo insertion in tissue) were noticed. A platinum electrode coated with overoxidized PPy with superoxide dismutase was tested as a superoxide anion [242]. Details of transport through the membrane were discussed, resulting in doubts regarding the selectivity of this electrode vs. hydrogen peroxide.

Several overoxidized ICPs incorporated in an acrylate-based solid polymer matrix were directly electrosynthesized [243]. Overoxidation destroyed the electronic conductivity of the ICPs and provided an ion-exchange polymer. The ionic conductivity of this composite was higher than that of the solid polymer matrix.

Overoxidation of an ICP or an ICP-containing composite can yield a material (in most cases a coating) serving various purposes:

1. Protection against electrode fouling;
2. Provision of permselectivity;
3. Action as a host or cover for immobilized reactants (in most cases, enzymes);
4. Provision of the material for molecular imprinting.

Function 1 is rather general and will be mentioned in passing whenever authors considered this property noteworthy. Functions 2–4 will be addressed in the following sections in this order, starting with the most common ICPs. Infrequently studied ICPs are collected at the end of this chapter.

PANI can be transformed by electrochemical overoxidation into a permselective membrane [244]. Permselectivity vs. anions was attributed to incorporated anions, not to functional groups generated by overoxidation [133]. Surface availability might play a role in the performance of permselective membranes based on overoxidized ICPs [245].

Permselective properties of overoxidized and, thus, electronically insulating PPy were reported first in 1992 [127]. Presumably, these properties were employed when coating a carbon paste electrode with overoxidized polypyrrole and polyvinylpyrrolidone films for the determination of phenol [246]. A film composed of overoxidized PPy and multi-wall carbon nanotubes on a carbon microfiber electrode enabled selective catalysis of dopamine oxidation for in vivo analytical application [247]. The permselectivity of overoxidized PPy on glassy carbon for dopamine and ascorbate have been compared, and an influence on the volume of counter anions present during PPy preparation has been noticed [248,249]. A glassy carbon electrode modified with a composite of overoxidized PPy and graphene was used for amperometric determination of dopamine, even in the presence of a large excess of ascorbic acid [250]. A graphite electrode modified with nanofibrous overoxidized PPy showed high selectivity for dopamine determination [251]. A coating with overoxidized PPy on carbon fiber microelectrodes was used with fast-scan cyclic voltammetry (300 V·s^−1^) for dopamine determination with high rejection of ascorbate and dihydroxyphenylacetic acid [252]. This electrode showed three times the sensitivity to dopamine than a comparison electrode coated with Nafion^®^. A carbon ceramic electrode modified with overoxidized PPy showed high sensitivity for the electro-oxidation of folic acid; selectivity was not addressed [253]. A graphite electrode modified with overoxidized PPy was evaluated for the determination of five sulfonamides [254].

Highly boron-doped diamond microfibers coated with overoxidized PPy served as amperometric sensor electrodes for dopamine with high rejection of ascorbic and 3,4-dihydroxyphenylacetic acid [255]. Compared to carbon microfiber-based electrodes, the detection limit was lower by one order of magnitude; because of the inertness of the diamond material towards adsorption, electrode fouling was reduced, resulting in stable behavior for at least two months. Another option is the use of a composite of gold nanoparticles and overoxidized PANI instead of PPy, with gold nanoparticles supporting dopamine sensing in the presence of ascorbic acid [256].

Vanillin could be determined selectively in commercial samples with a glassy carbon electrode coated with overoxidized PPy [257]. Electropolymerized films of poly[1-(2carboxyethyl)pyrrole] were easily overoxidized, yielding a film with high permselectivity for dopamine in the presence of excess ascorbate [258]. The potentiometric properties of PPy and overoxidized PPy films, particularly cation selectivity, were compared. Taking into account the counter anions present during formation, overoxidation, and potentiometric measurements, options of templating for enhanced selectivity were suggested [259]. The use of overoxidized PPy in potentiometric detectors for ion chromatography of aqueous samples was studied [260]. Ultra-thin, porous films of overoxidized PPy were prepared electrochemically on microdisc electrodes and characterized [261]. Additional XPS results [262] and results suggesting a nanometer detection limit for dopamine have been made available [263]. Permeability for H_2_O_2_ and surface coverage of overoxidized PPy films on a platinum substrate was determined simultaneously with a newly developed method enabling better insight into the influence of preparation conditions [264]. The precoating of a bare platinum surface with platinum black favored uniform nucleation of PPy, and the total polymerization charge was reported as a measure of the film thickness. The performance of overoxidized PPy and poly(*m*-phenylenediamine) films as permselective coatings for H_2_O_2_-sensing electrodes was compared, and the latter film performed better [265]. Amperometric determination of isoniazid was possible with a glassy carbon electrode modified with a coating of overoxidized PPy, but selectivity was not addressed [266]. Thiolated and overoxidized poly(*m*-phenylenediamine) subsequently coated with a film of bismuth on a glassy carbon electrode was used successfully for the simultaneous detection of Cd^2+^ and Pb^2+^ ions [267]. A glassy carbon electrode coated with overoxidized PPy with dodecyl sulfate as a counter anion showed high dopamine selectivity, in particular, vs. ascorbate anions [268]. Preaccumulation helped in lowering the detection limit. Overoxidized films of PPy turned out to be helpful in the anodic stripping voltammetry of several heavy metal cations with a mercury film electrode [269]. With this method, Cd^2+^ and Pb^2+^ ions were determined with a bismuth-covered glassy carbon electrode coated with Nafion^®^ and overoxidized 2-mercaptoethanesulfonate-tethered polypyrrole [270]. Dopamine and ascorbate were determined simultaneously amperometrically with a carbon fiber electrode coated with overoxidized poly(*o*-phenylenediamine) (i.e., poly(1,2-phenylenediamine)) prepared electrochemically with dodecyl sulfate as a counter anion [271]. Ascorbic acid, dopamine, uric acid, and tryptophan were determined simultaneously with an amperometric sensor based on a coating of overoxidized polyimidazole with electrochemically embedded gold nanoparticles on a glassy carbon electrode [272].

The overoxidation of a PPy layer in the presence of various counter anions resulted in enhanced sensitivity towards the amperometric detection of dopamine [273]. Microscope pictures revealed a flattened morphology of the PPy after overoxidation, and the still-present 3D polymer with carbonyl and carboxylic acid functions (presumably formed during overoxidation) supported the diffusion of dopamine. PPy prepared electrochemically with aszophloxine as a counter anion subsequently overoxidized was identified as a sensor material for simultaneous amperometric determination of dopamine and acetaminophen [274]. In the same way, these authors obtained a sensor material for methyldopa when using Titan yellow as a counter anion [275]. Overoxidized PPy prepared with 4-*N*-pentylphenylboronic acid as a counter anion increased affinity towards diols [276]. This was subsequently examined in the electrochemical amperometric determination of dopamine and in studies on the adhesion of bacteria. A gold electrode coated with overoxidized PPy containing nuclear fast red as a counter anion served as an amperometric sensor for the simultaneous determination of ascorbic acid and methyldopa [277].

PPy prepared with sulfosalicylic acid as a counter anion and selective ligand was used after overoxidation for the determination of Cu^2+^ ions with high selectivity and sensitivity [278]. The same approach with nitroso-R as a counter anion instead was reported [279]. A further option employing 4,5-dihydroxy-3-(p-sulfophenylazo)-2,7- naphthalene disulfonate as a counter ion was reported [280]. With the counter anion 2(2-pyridylazo)chromotropic acid in overoxidized PPy, a material for the accumulation of Pb^2+^ ions suitable for subsequent stripping voltammetry was prepared [281]. Overoxidized PPy on a gold electrode was applied in Cr (VI) determination after preconcentration [282]. A “memory” effect increased the detection limit in subsequent determinations.

A glassy carbon electrode modified with a composite of overoxidized polyimidazole/graphene oxide enabled the simultaneous determination of ascorbic acid, dopamine, uric acid, guanine, and adenine [283]. A similar simultaneous determination of ascorbic acid, dopamine, uric acid, and tryptophan was possible with an amperometric sensor based on a glassy carbon electrode modified with copper nanoparticles painted over a coating of overoxidized poly(3-amino-5-mercapto-1,2,4-triazole) [284].

Electropolymerized and overoxidized poly(*N*-acetylaniline) with β-cyclodextrin chemically attached to it formed an inclusion complex of cyclodextrin with cocaine determined with impedance measurements [285].

The overoxidation of PPy intended for use as a permselective barrier in an amperometric sensor in a NaOH/methanol solution yielded an overoxidized and partially hydroxylated and methoxylated PPy with spatially different distribution of these functionalities [286]. The ion exchange capability of overoxidized PPy was employed in a monoamine neurotransmitter detector for liquid chromatography [287].

The reduction of the background current noticed with an amperometric enzymatic biosensor with formate dehydrogenase embedded in PPy for use as a formate sensor caused by a reaction intermediate (NADH) was achieved by the overoxidation of PPy [288]. L-lysine α-oxidase immobilized on a platinum electrode by co-crosslinking subsequently covered with overoxidized PPy enabled the detection of lysine as an amperometric biosensor [289]. Overoxidized PPy was used in a sensor for various neurotransmitters. The high electronic insulation of the polymer and the uniform porous structure provided suitable diffusion conditions for electrochemically immobilized DNA [290]. DNA immobilized in the micropores of overoxidized PPy increased rejection of ascorbate and uric acid and enhanced preference for dopamine and epinephrine in an amperometric sensor [291]. The permselectivity of overoxidized PPy for various neurotransmitters was examined, and the helpful antifouling capability when preparing actual sensors was noted [292]. DNA immobilized in a template of overoxidized PPy on carbon fibers was used in a simultaneous determination of serotonin and dopamine with high selectivity and sensitivity, even in the presence of a large excess of ascorbic acid as a common interferent [293]. Glassy carbon covered first with multiple layers of betamethasone as a chiral recognition ingredient and coated with overoxidized PPy and graphene nanosheets was employed in a simultaneous quantification of mandelic acid enantiomers [294]. With a carbon paste electrode coated with overoxidized PPy, tryptophane could be determined by stripping voltammetry, even in the presence of a 15-fold excess of tyrosine [295]. PPy electropolymerized from an ionic liquid was overoxidized and examined with respect to permselectivity, and further immobilization of glucose oxidase for use in the coating as a glucose sensor was achieved by simple electrosorption [296].

Glucose oxidase was immobilized on gold nanoparticle-decorated overoxidized PPy for use as an amperometric glucose biosensor [297]. Using CuO instead of gold a sensor material for nonenzymatic determination of glu-cose was obtained [298]. On a glassy carbon electrode, a composite of overoxidized PPy and gold nanoparticles was used as a platform for a label-free impedimetric immunosensor for anti-transglutaminase [299]. Composites of ICPs with various mostly inorganic materials have been examined as sensor materials. The overoxidation of PPy in a nanocomposite of PPy and graphene for the detection of adenine and guanine was recommended for reducing the background current frequently found to be disturbingly high elsewhere [300]. A composite of gold nanoparticles and overoxidized PPy formed on a carbon ceramic electrode initially coated with MWCNTs showed high selectivity in the simultaneous determination of catechol and hydroquinone in water [301]. Glassy carbon modified with a layer of directed multi-walled carbon nanotubes and overoxidized PPy showed remarkable electrocatalytic activity in the oxidation of DNA bases [302]. This, in turn, enabled the development of a procedure for the rapid determination of DNA methylation status.

Glassy carbon coated with several layers of biotin-loaded overoxidized PPy and nanosheets of reduced graphene oxide was studied as an enantioselective electrochemical sensor for R-mandelic acid [303]. No interference of S-mandelic acid was observed. In another approach to an enantioselective sensor, overoxidized PPy with dexamethasone as a chiral recognition element on a graphene-modified glassy carbon surface was used [304]. General features of overoxidized PPy in chiral discrimination of several analytes were discussed in [305].

The electrochemistry of antibody-modified PPy and its interaction with an antigen with the ICP in neutral, oxidized, and overoxidized states was studied [306]. Overoxidized PPy decorated with gold nanoparticles on a screen-printed electrode was used to build up a label-free immunosensor for the determination of human serum immunoglobulin G [307].

Graphite modified with overoxidized PTh was examined as a sensor material for the amperometric determination of propham, showing high selectivity [308]. A similar approach with PEDOT as the overoxidized ICP and uric acid as the analyte was reported by these authors [309]. Experimental parameters for the electropolymerization of high-quality overoxidized PEDOT films were determined [310]. During formation of vesicles containing gold nanoparticles and a wall of PTh overoxidation of thiophene units yielding thiophene sulfone, units resulting in a vesicle wall with both hydrophobic and hydrophilic subunits (an amphiphilic polymer) were noticed [311]. Nanosized palladium clusters were electrodeposited in PEDOT [312]. Electrocatalytic activity for hydrogen sorption was less pronounced for a composite prepared with overoxidized PEDOT.

Overoxidized PEDOT on a screen-printed carbon electrode was successfully applied in the determination of submicromolar dopamine concentration without interference from a 1000-fold excess of ascorbic acid [313].

Several ionophores immobilized in overoxidized PPy enabled the joint quantification of potassium, ammonium, and sodium by impedance measurements [314].

The enantioselective uptake of glutamic acids by carbon fibers coated with overoxidized PPy packed into a column and the subsequent release by applying a suitable electrode potential were realized [315]. The controlled release of glutamate from overoxidized PPy used as a molecular switch was reported [316].

A glassy carbon electrode modified with an overoxidized PPy–gold nanoparticles composite subsequently covered with electrostatically attached transglutaminase antigen and, finally, capped with bovine serum albumin was developed as an immunosensor for the electrochemical determination of antitransglutaminase antibodies [317]. A platinum wire coated with overoxidized PPy and Nafion^®^ with attached glutamate oxidase was used as a microelectrode for the detection of alanine aminotransferase [318]. A cholesterol biosensor with cholesterol oxidase entrapped in PPy for use in a flow system utilizing PPy overoxidation to provide anion-exclusion capabilities to PPy formed minimized interference of other electroactive species (e.g., uric or ascorbic acid) [319]. An amperometric bilayer sensor of overoxidized PPy with entrapped lactate oxidase coated with poly(*o*-phenylenediamine) was tested in the determination of L-lactate [320]. In an ethanol biosensor, overoxidized PPy was used for the immobilization of alcohol oxidase [321]. The use of poly-*o*-phenylendiamine instead of PPy resulted in lower selectivity. Simultaneous determination of ethanol and glucose with a dual-amperometric biosensor with a dual gold electrode coated with overoxidized PPy, glucose oxidase, and alcohol oxidase immobilized with bovine serum albumin and glutaraldehyde was developed and tested successfully [322]. A glucose sensor with both glucose oxidase and horseradish peroxidase entrapped in electropolymerized PPy based on H_2_O_2_ reduction at *E*_Ag/AgCl_ = 0.15 V was tested [323]. At *E*_Ag/AgCl_ = 0.7 V operates based on H_2_O_2_, oxidation had lower sensitivity. The stability of the electrode in both modes of operation was not examined. The electropolymerization of pyrrole–glucose oxidase mixtures on a platinum electrode yielded a highly sensitive glucose biosensor [324]. Noticeable interferences were suppressed by adding an inner layer of overoxidized PPy or Nafion^®^ for better selectivity. The latter inner layer resulted in a better selectivity performance.

**Figure 21:**
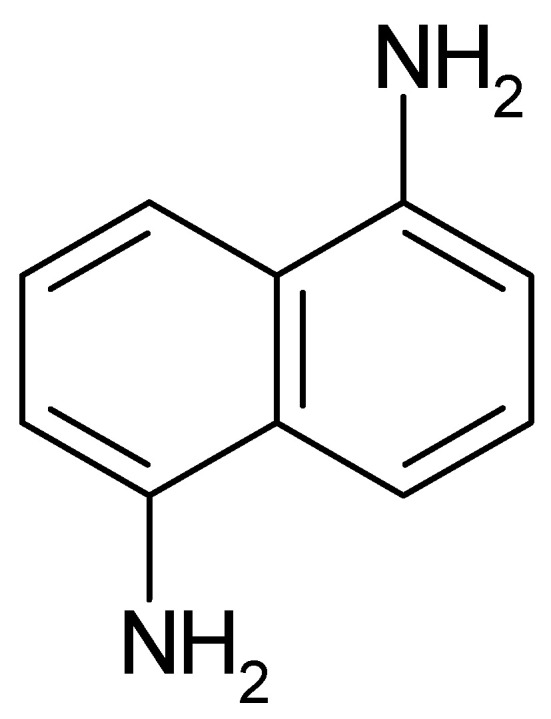
1,5-diamino naphthalene.

The overoxidation of PPy as a part of a bioactive and electrically conducting hydrogel used as a biotransducer for glucose eliminated the background current, similar to observations noticed above [325]. The stability and performance of a PPy film with dodecyl sulphate as a counter ion and entrapped glucose oxidase in an amperometric glucose sensor was improved by deliberate overoxidation at the expense of a slightly decreased glucose response [326]. A highly selective amperometric glucose sensor based on glucose oxidase immobilized in overoxidized PPy has been reported [327,328]. An enzymatic sensor for glucose and cholesterol employing a layer of overoxidized PPy with the corresponding enzyme (glucose or cholesterol oxidase) entrapped therein and, finally, coated with nonconducting poly(*o*-phenylenediamine) was assessed and compared with a single layer system (without the additional topcoat) [329]. The performance of the bilayer system was slightly better. A similar bilayer system based on overoxidized PPy and poly(1,5-diamino-naphthalene; (the term poly(1,5-diamino-polynaphthalene) is obviously incorrect) (see Figure 21) was suggested by these authors for cholesterol determination [330]. Other isomers of diamino naphthalene yielded polymers resulting in poorer performances.

PPy was proposed as sensor material for the detection of OH radicals [331]. The overoxidation they cause results in reduced electronic conductivity of the polymer, which was used as a measure of the amount of radicals that reached the polymer surface.

An electrochemically oxidized graphite electrode modified with overoxidized poly(pyrrole-3-carboxylic acid) was studied as a disposable dopamine sensor, showing sufficient selectivity against common interferents [332].

Electrochemical overoxidation by controlled application of higher electrode potentials to a molecularly imprinted ICP layer may be used to remove the molecular template [55,333,334]. A nanostructured and molecularly naproxen-imprinted layer of PPy showed enhanced selectivity in naproxen determination after carefully controlled electrochemical (CV) overoxidation [335]. Electropolymerized and subsequently overoxidized molecularly imprinted polypyrrole films provided a higher enantioselective recognition of L-aspartic than D-aspartic acid, and their suitability in an EQCM was demonstrated [336]. The electrode-potential-induced enantioselective uptake of amino acids was studied with electrochemically overoxidized molecularly imprinted PPy [337]. The treatment of PPy-coated microspheres with an aqueous solution of 0.1 **M** NaOH (effecting overoxidation) [332] removed the imprinted cells of Escherichia coli by curing and dedoping through providing a specifically and efficiently binding sensor option [338]. This procedure has been applied also with other analytes and PPy [336,339]. Molecular recognition towards bile acid was afforded to overoxidized PPy by imprinting it with taurocholate, yielding a sensor both highly sensitive and selective [340]. Ultra-thin films of overoxidized PPy molecularly imprinted with various purines showed significantly enhanced sensitivity for adenosine, in particular, when adenosine 5′-triphosphate was used for imprinting [341].

The selectivity of molecularly imprinted overoxidized PPy was applied in the microextraction of ibuprofen [342]. Similar approaches have been employed for the extraction of salicylate [343] and of sulfonamides [344]. The molecular selectivity of a molecularly imprinted and overoxidized copolymer of pyrrole and indole-2-carboxylic acid was studied for the co-extraction of acidic, basic, and neutral drugs [345]. Molecularly imprinted and overoxidized PPy has been verified as a sensor material for the amperometric detection of sulfadimethoxine [346,347]. Molecularly imprinted and overoxidized PPy formed on MOF-derived porous carbon–carbon nanotubes composite and Prussian blue nanocubes provided enantioselective recognition of L/D-cysteine [348]. Gold nanoparticles embedded in overoxidized PPy molecularly imprinted with L-cysteine showed a better chiral recognition of cysteine enantiomers because of Au–S interactions absent in the coating without gold nanoparticles [349].

Molecularly imprinted overoxidized PPy was used in an electrode for nicotinamide analysis [350]. Preparation conditions and parameters for molecularly imprinted overoxidized PPy modified further with platinum nanoparticles to be used as a sensor for vardenafil detection were examined and defined [351]. PPy molecularly imprinted with L-tryptophan and subsequently overoxidized was successfully used in the enantioselective recognition of tryptophan enantiomers [352].

Instead of using PPy films, colloids of PPy were suggested [353] and reviewed [354]. The reported examples included various amino acid enantiomers [355]. In most cases, the species to be detected later was already involved in the imprinting. The possibilities of detecting species only similar (and not identical) to the one used during imprinting were examined with overoxidized PPy colloids and L-lactate used for imprinting [356]. The application in the enantioseparation of amino acids was tested.

The recognition of Gram-negative and -positive bacteria was achieved with overoxidized PPy imprinted during potentiostatic PPy deposition in a solution also containing the monomer and bacteria [357]. Bacterial surface chemical structures were exactly transferred to the ICP film at a molecular level. Similar observations extending the range of detectable bacteria were reported elsewhere [358].

The electrode-potential-triggered release of L-glutamate from molecularly imprinted overoxidized PPy was realized [359].

The incorporation of chelating ions with metal specificity into an ICP film may be an option for trace metal determination [360]. Overoxidation was examined (as already discussed above for a different sensor approach) as a way to reduce the large background current [361]. Overoxidized sulfonated PPy obtained by electropolymerization was examined as a material for the solid-phase microextraction of trace levels of nickel and cadmium ions [360]. This application of method and material was extended to further cations and anions [362].

Overoxidized PEDOT:PSS was incorporated into an organic bioelectronic ion pump [363].

Molecularly imprinted and overoxidized poly(indole-3-acetic acid) deposited on multi-walled carbon nanotubes immobilized on graphite enabled the enantioselective detection of D- and L-aspartic acid [364].

Overoxidized polydopamine used for embedding silver nanoparticles provided capabilities similar to those of overoxidized PPy for sensing *p*-nitrophenol [365]. Such overoxidized polydopamine film was used in an amperometric dopamine sensor [366]. Overoxidized polydopamine subsequently modified with 3,4,9,10-perylenetetra-carboxylic acid was used as both sensitive and selective sensor material for the simultaneous determination of ascorbic acid, dopamine, uric acid, xanthine, and hypoxanthine [367].

The overoxidation of electropolymerized poly(1-naphthylamine) yielded a sensor material for the selective determination of dopamine in the presence of a large excess of ascorbic acid [368].

Longitudinally unzipped, multi-walled carbon nanotubes incorporating overoxidized poly(*p*-aminophenol) on a glassy carbon support enabled the simultaneous determination of dopamine, uric acid, and tryptophan [369]. Poly(alizarin red S) overoxidized with differential pulse voltammetry on a graphite electrode was used for the simultaneous determination of hydroquinone and catechol [370].

The loss in electronic conductivity observed with ICPs upon overoxidation was utilized in the patterning of a nanostructured free-standing PEDOT-based bilayer film [371]. Chemical overoxidation of PEDOT:PSS with sodium hypochlorite applied spatially resolved with an inkjet printer was a step in a process for preparing free-standing nanofilms [372].

Nanocrystals of cobalt hexacyanoferrate with uniform shape and size were prepared on a ceramic carbon electrode modified with overoxidized PPy in the presence of ethylene diamine tetraacetic acid [373]. They showed high catalytic activity and improved stability in the electro-oxidation of hydrazine.

A mathematical procedure was developed for the evaluation of CVs obtained with overoxidized PPy in sensor applications with overlapping current peaks [374].

## 4. Miscellaneous Observations

In a two-electrode arrangement (typically a battery or a supercapacitor, but also an actuator with some counter electrode) voltage (i.e., the difference between the electrode potentials of both electrodes) has been applied without the control of a particular electrode potential [375,376,377,378]. A device with two PPy electrodes was examined, and a maximum cell voltage of 1.5 V was found to be safe in terms of avoiding overoxidation. Electropolymerized nanostructured films of aminophenyl porphyrin showed a loss in electronic conductivity similar to changes observed with PANI during its transition from emeraldine to pernigraniline [379].

Overoxidation of the ICP employed as channel material in an organic electrochemical transistor was used to afford permanent changes of material properties [380].

The overoxidation of PEDOT used in an all-polymer electrochemical transistor with associated conductivity loss was employed in a “write-once read-many-times” application [381]. The preparation of high-redox-capacity PEDOT:PSS electrodes showing no overoxidation when 100 V were applied was described, although evidence for this claim was not provided.

Overoxidation by air (presumably dioxygen) has been noticed as a problem with ICP-based heterojunctions, diodes, and capacitors prepared using a lithographic approach [382,383]

Powders of PPy and PEDOT have been prepared by chemical oxidation with ammonium persulfate for use as radical scavengers [384,385]. Scavenging capability, as well as electronic conductivity, decreased when more oxidant was used in the synthesis. This was attributed to overoxidation monitored with infrared spectroscopy. The selective uptake of some amino acids was greater with overoxidized PPy than with not-overoxidized, but the observations were rather mixed [386].

To avoid issues with PPy overoxidation in a polymer battery, the authors switched to PEDOT as a positive electrode material [387].

Because of the apparently significant importance of overoxidation, sometimes the high stability of an ICP versus overoxidation is claimed without providing any evidence [388].

**Figure 22:**
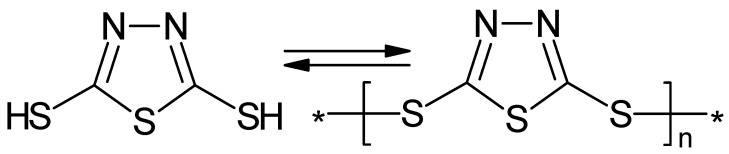
2,5-dimercapt-l,3,4-thiadiazole.

Overoxidation of electrochemically active (but not electronically conducting) polymers, such as 2,5-dimercapt-l,3,4-thiadiazole (Figure 22), were reported, but they are beyond the scope of this contribution [389].

## 5. Conclusions

In many applications of ICPs, overoxidation mostly by electrode potential excursions results in performance losses for the ICP in its particular function, such as irreversibly diminished electronic conductivity, reduced charge storage capability, and changed morphology. Various options to avoid this are either obvious (closer electrode potential and cell voltage control) or have been suggested (forming copolymers, adding radical scavengers to electrolyte solutions, etc.); in any case, poorer performances of electrochemical systems based on ICPs may be attributed to overoxidation as a likely cause. According to the rich evidence surveyed above, it can be stated that overoxidation is inseparably connected with the redox activity of ICPs because the oxidized form of every ICP is basically susceptible to chemical, particularly nucleophilic, attacks from the chemical environment. Thus, from the oxidized state, an ICP may—as desired—return by reduction to the neutral form or—as not desired—by chemical reactions into overoxidation or degradation products. Consequently, the definition of a clear-cut overoxidation electrode potential is basically impossible. All reported data may be interpreted in relative terms, in which potential range reversible redox activity dominates and overoxidation takes over.

There is, quite to the contrary, a large amount of scientific reports wherein ICPs overoxidized in a controlled manner have been applied in sensors, electrocatalysts, and further functions. A better understanding of the beneficial effects of overoxidation will be helpful in the development and optimization of sensors and further applications. It might also help in controlling the unwelcome effects of overoxidation in other applications better. The occasionally noticed expulsion of counter anions during overoxidation sheds some doubt on the role of ionic species (frequently called dopants) incorporated in overoxidized ICPs and, apparently, still present after overoxidation.
